# Address before the Graduating Class of Baltimore College of Dental Surgery, March 12, 1868

**Published:** 1868-04

**Authors:** Henry R. Noel


AMERICAN JOURNAL

OF

DENTAL SCIENCE.

Vol. 1. THIRD SERIES—APRIL, 1868. No. 12.

### ORIGINAL COMMUNICATIONS.

### ARTICLE I.

### *Address before the Graduating Class of Baltimore College of Dental Surgery, March 12, 1868.*

By Professor Henry R. Noel, M.D.

[We present to our readers the admirable address of Professor Noel. To adapt it to our limited space we have been obliged to omit many paragraphs. We do this with the less regret, because at the solicitation of of the class, it has been printed, as delivered, and those who wish to see it in full, can obtain copies by addressing Dr. Noel or any other member of the Faculty.] Eds.

Gentlemen:

To-night our collegiate work closes. Our mutual relations are about to be dissolved, and the ties binding professors and students severed forever. It has ever been deemed appropriate that upon such occasions, some one of your professors should, on behalf of the college, tender you a farewell and give advice and encouragement.

This honor has devolved upon me and places me in a position somewhat embarrassing. A medical man—I cannot give you advice about dentistry. The summers I have numbered, are too few, for me to assume the role of the wise man of the world, and give you advice based upon practical experience.

There is, however a ground upon which we all can meet; there is a subject appropriate to the occasion. It is the

THEORY OF SUCCESS IN LIFE.

This theory is not genius—it is not the possession of a dazzlingly brilliant intellect. It is not so much the *original*, as it is the *acquired* power of mind; and it is found in the continuous application of mental force. It is perseverance—it is untiring work. A homely theme but one that comes searchingly near to the hearts of many, if not of all, here to-night. Certainly it comes very near indeed to us.

"We have *not* been born great;" "we have never had greatness thrust upon us;" and there remains you know but one other solution of the position, and that is

"To achieve it."

Achieve success—it is no boy's play. We should enter this struggle with no misconceptions of its extent; no misconceptions of its true meaning; but fully comprehending that in its height, breadth and depth is contained the very sum of our existence; the sum of our happiness—the realization and fruition of our hopes and our dreams; or hopes blighted—blackened—blasted—ruined. We must enter with all our mind, soul, and strength,—with no diffusion, but an intense concentration of mental force; for the struggle is the battle of life and we are well aware that the "laurel wreath" rests only upon the brow of him, who has known work in the bitterest acceptation of the term. Our ideas upon all subjects should be clear and definite, and this is especially true of our professional careers, for false impressions in early life, often lead to miserable failures.

Now, we shall tell you through what you must pass to success. But we shall point you out no gilded career, no rose-colored path to greatness. The world has nothing of that kind for us, but it has another kind of road even to bare comfort no even graded level—no tessellated pavement ; but rough—rugged, rocky ;—tortuous, twisting, turning ; ascending, descending, broken and uneven ; with many a sharp angle and jutting crag athwart our path ; with many a toppling fragment over head and yawning chasm beneath. Often worn and weary with much to dishearten, much to depress, with many a heart-ache and many a throbbing brow ; we must tread the narrow path of self-denial and continuous labor.

The world has for us no honors, save those we wring from it by our own efforts. Through dreary months of anxiety and suspense ; through long years of slow toil we must walk this path, and see the realization of our hopes yet beyond us—and often simulating the mirage of the desert, receding as we advance. We shall know well the yearning of the weary brain for rest ; we shall know well the burning of the heart for sympathy in its hours of tension and strain, when encouragement falls like a heaven-sent shower upon a parched and thirsty earth.

You wish bread—you wish success—you wish honorable position. The marts of this world contain them all—but the currency it demands is coined brain—intellectual labor. There is no escape ;—there is no reprieve ; it is not optional—it is imperative, and a inexorable necessity has decided the future for us. The sentence once spoken at the gates of Eden, has reached down through centuries and crossed our path. The curse of the race is fulfilled in us, as it was in our first parents ; the decree is fixed, the law immutable ; and to it finite man must humbly bow. Man has been great—man can be great. What man has done—man can do, but only by self-conquest—self-denial, study, application, labor.

"The dignity of labor," a theme so very eloquently handled by our would-be orators, we do not appreciate.

We have known labor, intense labor, prolonged labor, but we have signally failed to perceive its dignity ; in fact we see in it only the primeval curse. Labor was never intended as a blessing for man—at least we are not so taught in Genesis. Yet only by labor can true success be attained, it hasnever been attained otherwise, and through this avenue alone can *we* attain it. Its relation to us and to the world are such, that, it becomes the means of obtaining an end. The end is grand, but the means are not so.

The end is success—it is noble and worthy of any sacrifice, and we must make the sacrifice—a continued sacrifice of self. Now this is the plain truth—unadorned, unembellished—you must work—there is no escape. He who lingers in pleasure's paths, or stands in idle dalliance with fashion's votaries, is lost, lost, lost.

The true theory of success being work, what is the true theory of working successfully ? We answer—to systematize—to concentrate. To obtain definite results, to obtain these in their highest perfection, we must acquire the habit of prolonged, continuous and intense concentration of mental power.

We must learn to concentrate the whole intellectual force upon a given point and hold it there, till like the convex lens converging and concentrating the diffused rays of the sun, we fuse as it were the dross of error and obtain imperishable truth. Throw mental light upon dark subjects — dispel the mists of uncertainty ; — make the obscure bright and patent. One great element of success is the acquired habit of using the powers of the mind upon the convex lens plan of convergence—concentration.

Carlyle, we think it is, who eulogizes the beaver intellect; that intellect never brilliant, never dazzling, but which slowly, surely and inevitably attains its ends, secures its prize. Never daunted—never faltering—never wavering—never crushed—always working—never despondent—always hopeful : they toil onward and upward —higher and yet higher—until upon the summit they watch the mists dissolving below them, and from their exalted position looking down, they reap the reward of concentrated, persevering, untiring effort. There is no other road—there is no success worthy of the name, that is not obtained by coined brain.

Thus far we have arrived at the conclusion that success implies an invariable antecedent, and that antecedent is labor. Now we wish to prove that labor is the law of developement both physical and mental.

We shall begin with the muscular system, and shall show you how more muscle, and more muscular power are acquired in direct ratio to the amount of exercise—amount of labor. We shall then reason by analogy from this to the nervous system, drawing aid from observation and practical experience, as we proceed. And we shall endeavor to prove that the law for one obtains in the other—and that the nervous system also, is developed by exercise—by labor.

The muscular system is the seat of muscular force, physical power; the nervous system is the seat of mental force, intellectual power; if therefore we succeed in demonstrating that labor is the law of developement for the one, by analogy it will apply to the other also, the deduction therefore will be that muscular and mental power alike depend upon exercise of their respective systems. Now there is not one thoughtful student present to-night, who does not believe in the entire truth of this statement.

As a practical fact too you know it. You know that the athlete and the victor in the prize-ring, attain their superiority by a rigidly systematic and protracted exercise of the muscular system. The degree of success is in direct ratio to the developement of that system, and the acquired habit of using that developement. The result is the production of muscle and the scientific application of its action.

It is simply a correlation of force; the human vital force in the muscle is converted into motion; so much muscular tissue is destroyed, and so much physical force realized in return. The relations are fixed—the factors are invariable; the law is exercise, the result is developement.

Look at the brawny arms of the blacksmith. Look at the huge muscles of the coal heavers. But your own experience is sufficient attestation of this, though very few seem to thoroughly understand the application of experience in proof of this law.

Which of you, gentlemen, first swung with ease, over your heads your 25 pound dumb bells? Which of you, but began with much lower figures, and gradually developed the power as you fulfilled the law of your physical being developement by labor? Labor being the law, what is the penalty of a violation of this law? What is the penalty of muscular idleness? You know it well. Loss of size; loss of control over the muscles, impaired vigor; impaired power; a slow decay; a species of caries of the whole animal physique. One example will suffice. You have a fractured arm—you carry your hand and arm splinted and slung up for from four to six weeks:—you take it down—remove the dressings, splints, &c.; the bone has knit—the limb is apparently sound: true; it is emaciated and looks the invalid. Attempt to straighten it—you cannot do it; the muscles stubbornly refuse to obey your commands. You have kept that arm quiet too long,—you have violated the laws of muscular action and muscular integrity. You have disobeyed the commands of nature—and nature's children in their turn disobey you.

How do you regain control over that limb? How make it again your slave? By *learning obedience*, by following the prescribed law—exercise. Surgeons call it "passive motion." Do you not see how *very rigidly* this law is enforced?

Idleness is therefore not merely non-acquisition, it is direct and absolute retrogression.

Can we apply this law to the nervous system, the seat of all intellectual phenomena? It is eminently true of this system, for only through the nerves can the will act upon the muscles, and the education and developement of the muscular, presupposes and involves a species of exercise of the nervous.

The brain we know to be the great receiving and dispatching depot—the great magazine for storing up impressions; the great workshop—the grand laboratory for the evolution of mental force; and with its telegraphic threads or cords, which we call nerves, is in direct communication and relation with the whole body and external world. All moral, emotional and rational phenomena have their origin in changes of the substance of the brain. The brain is the seat of intellect.

We can acquire, by exercise, more muscular tissue and a better control over the tissue. Can we, by exercise, acquire more brain—can we acquire a better control over the brain?

Understand of course, that the brain is but the material instrument of that immaterial agent we call the *soul.* To answer the first question, would lead us into a physiological and metaphysical discussion, not suited to the occasion. But the latter we can answer boldly and clearly in the affirmative.

No one will deny, that by rigid training, by systematic exercise, the powers of the mind can be greatly increased, greatly developed, greatly intensified. What do we mean by education? Certainly not that ridiculously absurd idea of simply filling up the mind, as if it were a reservoir capable of holding exactly so much and no more, which when once filled was done with education. This is the almost universal idea, but an extremely fallacious one.

The Romans had a clearer idea than this. They did not consider mental training to mean cramming—filling; on the contrary with them it was *educating*, (from e—out of, and duco to lead or draw forth) and the idea is exactly correct—a drawing forth of the powers of the mind. Mental developement was but the drawing out and strengthening of the mental powers, according to the Roman idea, and we earnestly recommend you to adopt this idea.

The law of developement by labor obtains, the analogy between the two systems is therefore complete.

The mental athlete swings his intellectual dumb bells from the primary school to his college commencement, and then feels that he is only *beginning* to acquire some little skill in their use ; that he is indeed a tyro upon the boundary of the vast world of letters ; that work which is to tell upon the world has yet to be performed—his real labors have but begun. The collegiate training is but preparatory and at best but disciplines the mind for the struggle The true unfolding of the mental resources is by years of study.

We know the penalty of idleness ot muscle, what is the penalty of an idle brain ? The phenomena are sad—very sad. The mind relaxes its efforts ; the intellect looses its brightness ; fancy checks her flights ; imagination ceases to be vivid ; the judgment is no longer accurate ; and reason unhesitatingly accepts the most incredible statements, or rejects the most patent truths, being utterly impotent to grasp or handle, to weigh or appreciate the simplest arguments. Trains of thought there are not ; but a confused series of disjointed, disconnected and illogical fragments are found upon this arid waste, this mental Sahara.

Beware of this quiescence ;—beware of this lethargy of brain; this stagnation of intellect. The saddest of all sights are these poor, attenuated, emaciated, and dwarfed minds, these arrests of intellectual developement ; and we can almost see that there is also spiritual atrophy, a sort of eremacausis of soul.

"In the sweat of thy face shall thou eat bread, until thou return unto the ground."

We understand this now—It is the curse of barrenness upon the earth, and reflected back upon the human race it becomes the law of developement. Adam was cursed ;—

his whole race was cursed—this same curse rests upon your muscular system,—it rests upon your nervous system; it rests upon man's physical and upon his mental nature alike. This is the philosophical solution of the question;—this is why labor so inexorably preceeds and determines success. Therefore you must labor.

By labor you can advance—not to labor is retrogression. It is not optional. God has made the law imperative.

Achieve success. Success gives power, gives wealth, gives leisure, gives rest. Ambition is a sharp spur, but poverty is infinitely sharper and more deeply and steadily applied.

Remember that necessity is a bitter task-master, but one we may be called upon to serve, beware lest we fall into his hands unprepared, i. e. without proper mental culture. Many of us know full well this necessity—we have seen poverty in all its uninviting hideousness; we have seen it wring the heart, until its anguish was indescriable.

Lands, jewels, silks, clothing, the very shoes, and even the hair, woman's ornament, peerless woman's pride, sheared close and bartered for bread. Aye—more than this, we have seen this monster stretch forth its hand, and place its bony fingers upon the throats of its victims and hoarsely whisper in the ear—Starvation. And the cheek so hollow would sink yet more;—and the lip so blue with famine would turn yet darker, and upon the faces would settle that horrible expression of hopeless despair. This is no fancy sketch—the very breeze that comes from yon land, bearing on its wings the warm breath of spring, is also freighted with the cry for help, which rises from a crushed and a starving people. And *our homes* and *firesides* are there, there with that stricken people.

Oh! it is a hard, bitter struggle—this struggle with poverty. Brave must be the heart—unfaltering the courage; untiring the energy, if one shall combat poverty, and also desire to attain success. Many of you will need all we have stated, for you are to return to that section of our country where the songs of the harvest reapers are scarcely heard, for the fields are lying fallow; where the streets of the cities are silent, and grass is growing through the pavement; where the shops are tumbling down; where the wharves are going to decay; where the school-houses are empty; where the churches are unfilled; where the curse of poverty is upon the people, and the cup is being drained to its last dregs.

Now there is one more argument, or reason why you should "attain success" even at any sacrifice, it is one that comes from the homes you have left. And you owe this, to those you have left behind and are soon to rejoin; the circle gathered around the fireside to night in those distant homes thinking now of you.

From the dark lagoons of Texas,—from the untilled cotton fields of Georgia and Alabama, from the hills of Tennessee, from the rice lands of the Carolinas, from Virginia's blood-stained bosom, from all that country "bowed down beneath a weight of woe," over whose once fertile fields, the roughened ploughshare of a brother's hate, has run in war's red maddening carnival, rise the prayers of sisters, the prayers of brothers, the prayers of fathers and the prayers of mothers. And their hearts turning to-night, northward to Maryland, even as turns the Moslem pilgrim to the shrine at Mecca, they turn to Baltimore. Their pride, their love, their treasures, and in many instances their only hope for the future are here —here with us to-night.

Shall this appeal be unheeded? can we appeal to you by anything stronger than—*By that country, sad and desolate? By the charred remains of the burned cities. By the blackened ruins of once happy homes. By the hopes thus blighted and blasted. By the sister's worn and weary look of anxious toil. By the father's faltering step and furrowed brow. By the mother's sad, stricken face and earnest prayer. By that bitter poverty, which holds them in its merciless and ever-closing grasp.*

*By the graves of the dead, and the altars sacred to them in memory—be true to the laws of your being—attain success.*

By patience, courage, self-denial, and unceasing labor, acquire the power, and the means to aid in paying the price of their redemption from the servitude of this abject want. You can and you must attain success.

Many fail because they do not work. Fail because they never systematize. Fail because they never have definite aims. Fail because the first reverse disheartens. Fail because such rigid self-denial is required. But to us there must be no such word as failure,—there is too much at stake, we cannot afford to fail—unless life fails first.

Your professors extend to you the warmest feelings of interest and friendship. They will rejoice in your prosperity. They will sympathise in your adversity. They will deeply regret—should any one of you make a failure in life.

They earnestly hope that in future years your names will be known, as those of true scholars—true workmen, and characterized by scientific research and professional acumen; that your Alma Mater may be proud of her sons.

